# Alleviating Climate Change and Pollution with Nanomaterials

**DOI:** 10.3390/nano10020358

**Published:** 2020-02-19

**Authors:** Muralidharan Paramsothy

**Affiliations:** NanoWorld Innovations (NWI), 1 Jalan Mawar, Singapore 368931, Singapore; mpsothy@yahoo.co.uk

Nanoparticles can be utilized to extract carbon from air, dyes from water and sludge from waste, and are gradually emerging as useful for tackling threats to our planet’s health. Nanomaterials that can efficiently use carbon dioxide from the air, capture toxic pollutants from water and degrade solid waste into useful products, are being developed. In line with this theme, seven representative articles [1,2,3,4,5,6,7] have been published in this Special Issue:

As reported by Li et al. [1], a novel three-dimensional (3D) flower-like Ag/AgCl/BiOCOOH ternary heterojunction photo-catalyst was successfully constructed via loading AgCl nanoparticles onto BiOCOOH microspheres, followed by photo-reduction. The extraordinary photocatalytic capability was primarily credited to enhanced sunlight harvesting capability and the suppressed recombination of photo-induced carriers, originating from the synergy within the novel ternary heterojunction system. The trapping experiments demonstrated that H+, •OH, and •O_2_^−^ reactive species contributed to the degradation of pollutants. In work by Zhao et al. [2], a new polypyrrole-grafted magnetic compound (CoFe_2_O_4_@SiO_2_-Ppy) was successfully synthesized with a facile hydrothermal method under relatively safe conditions. CoFe_2_O_4_@SiO_2_-Ppy effectively adsorbed Hg^2+^ ions from water and exhibited excellent regeneration ability, dispersibility, and stability. CoFe_2_O_4_@SiO_2_-Ppy can be an excellent adsorbent for removing heavy metal ions from aqueous solutions.

In He et al.’s work [3], a simple and effective method for trace analysis of 4-nitrophenol was developed to protect water resources and food supplies. Graphene was used as a novel electrode modifier due to its many excellent properties, such as high specific surface area, upstanding electric conductivity and excellent electrochemical catalytic activity. Acetylene black paste hybridized the electrode and provided a favourable micro-environment for 4-nitrophenol, effectively accelerating the direct electron transfer rate of 4-nitrophenol at the electrode surface. This study led to the development of a high-efficiency electrochemical sensor for environmental analysis, with improved qualities, such as simplicity of electrode preparation, wide linear range, low detection limit, high selectivity, rapid regeneration, and long-term stability. As reported by Li et al. [4], Ag_2_CO_3_ nanoparticles interspersed-BiOCOOH heterojunction photocatalytsts were prepared by a facile procedure. In comparison with pure Ag_2_CO_3_ and BiOCOOH, Ag_2_CO_3_/BiOCOOH exhibited superior photocatalytic activity for the degradation of toxic pollutants under simulated sunlight. The synergy between Ag_2_CO_3_ and BiOCOOH not only expanded the sunlight absorption range but also promoted the separation of electron and holes, leading to the outstanding performance of Ag_2_CO_3_/BiOCOOH. This novel photocatalyst can be used to degrade refractory pollutants under simulated sunlight.

Chen et al. [5] reported the preparation of Fe_3_O_4_-Au magnetic nanocomposites using a well-developed Au seed deposition method. The increase of the quantity of the Au seeds attached to the surfaces of the Fe_3_O_4_ hollow microspheres resulted in Fe 2p peaks shifting toward a lower binding energy and Au 4f peaks shifting towards a higher binding energy. Pseudo-first-order kinetics were used to calculate the rate constant of 4-nitrophenol reduction, and the rate constants for the 4-nitrophenol reduction increased with increasing Au seed content. The Fe_3_O_4_-Au magnetic nanocomposites can be excellent recyclable nanocatalysts for the catalytic reduction of organic pollutants in the treatment of waste water. Work by Liu et al. [6] demonstrated the fabrication of pure FePt nanocrystals, followed by that of FePt-Ag nanocomposites by chemical seeding. The increase of the number of Ag seeds attached to the FePt nanocrystals’ surfaces resulted in shifts in Fe 2p peaks and Pt 4f peaks toward the lower binding energy and shifts of Ag 3d peaks toward the higher binding energy. Pseudo-first-order kinetics were used to calculate the rate constant of reduction of methyl orange and Rhodamine B. The catalysis performance of FePt-Ag nanocomposites remained almost consistent, even after at least six cycles, demonstrating the potential industrial application of FePt-Ag nanocomposites as highly efficient recyclable nanocatalysts for the reduction of various organic dyes.

Critically unearthed by Bruk et al. [7], palladium at the surface of the PdCl_2_–CuCl_2_/-Al_2_O_3_ nanocatalyst is present in the form of PdCl_4_^2−^, containing amorphous particles, while copper exists as crystal nanophases with the crystal structure of paratacamite (Cu_2_(OH)_3_Cl) and cuprous chloride(II) (CuCl_2_.2H_2_O). The interaction with CO and water vapour results in carbonyl complexes, which are intermediaries in carbon dioxide formation. Carbon dioxide is formed upon the cleavage of the palladium carbonyl complexes in the presence of preliminarily coordinated water and copper(I)-activated oxygen. The correct choice of an active metal, especially one in the most active state among the available forms of this metal, is crucial for designing the most efficient CO oxidation catalysts. Hybrid organic–inorganic materials that promise an advantageous combination of structural and porous characteristics are of particular interest.
